# Effect of aging on audiovisual integration: Comparison of high- and low-intensity conditions in a speech discrimination task

**DOI:** 10.3389/fnagi.2022.1010060

**Published:** 2022-10-28

**Authors:** Weiping Yang, Ao Guo, Hanyun Yao, Xiangfu Yang, Zimo Li, Shengnan Li, Jianxin Chen, Yanna Ren, Jiajia Yang, Jinglong Wu, Zhilin Zhang

**Affiliations:** ^1^Department of Psychology, Faculty of Education, Hubei University, Wuhan, China; ^2^Brain and Cognition Research Center, Faculty of Education, Hubei University, Wuhan, China; ^3^Applied Brain Science Lab, Faculty of Interdisciplinary Science and Engineering in Health Systems, Okayama University, Okayama, Japan; ^4^Department of Neurology, Wuhan No. 1 Hospital, Wuhan, China; ^5^Department of Psychology, College of Humanities and Management, Guizhou University of Traditional Chinese Medicine, Guiyang, China; ^6^Research Center for Medical Artificial Intelligence, Shenzhen Institute of Advanced Technology, Chinese Academy of Sciences, Shenzhen, Guangdong, China

**Keywords:** audiovisual integration, aging, stimulus intensity, speech perception, inverse effectiveness

## Abstract

Audiovisual integration is an essential process that influences speech perception in conversation. However, it is still debated whether older individuals benefit more from audiovisual integration than younger individuals. This ambiguity is likely due to stimulus features, such as stimulus intensity. The purpose of the current study was to explore the effect of aging on audiovisual integration, using event-related potentials (ERPs) at different stimulus intensities. The results showed greater audiovisual integration in older adults at 320–360 ms. Conversely, at 460–500 ms, older adults displayed attenuated audiovisual integration in the frontal, fronto-central, central, and centro-parietal regions compared to younger adults. In addition, we found older adults had greater audiovisual integration at 200–230 ms under the low-intensity condition compared to the high-intensity condition, suggesting inverse effectiveness occurred. However, inverse effectiveness was not found in younger adults. Taken together, the results suggested that there was age-related dissociation in audiovisual integration and inverse effectiveness, indicating that the neural mechanisms underlying audiovisual integration differed between older adults and younger adults.

## Introduction

In our daily lives, events convey information through different sensory modalities. Our brain is able to integrate different sensory information into a unique and coherent percept. For instance, when communicating with others, we recognize speech content by integrating visual and auditory speech information. This process is called audiovisual integration (Laurienti et al., 2006; Stein, 2012). The benefit of simultaneous (bimodal) presentation of visual and auditory stimuli over unimodal presentation has been repeatedly validated; for instance, the response time to audiovisual stimuli is faster than that to visual or auditory stimuli alone (Teder-Sälejärvi et al., 2005; Yang et al., 2015).

As we age, sensory systems and cognitive functions significantly decline (Bäckman et al., 2006; Liu and Yan, 2007; Grady, 2012; Mozolic et al., 2012). Audiovisual integration can bridge the gap between sensory and cognitive processing (McGovern et al., 2014). Numerous aging studies have compared audiovisual integration between younger adults and older adults. Some researchers employed a color-naming task with semantic stimuli presented under visual, auditory, and audiovisual conditions (Laurienti et al., 2006). The results showed that the reaction time to audiovisual stimuli was reduced in younger and older adults, with a significantly greater audiovisual gain in older adults. Similarly, on a simple discrimination task with meaningless stimuli, older adults also exhibited enhanced audiovisual integration (Peiffer et al., 2007). To obtain a clearer and more complete understanding of the neural changes associated with audiovisual processing during aging, subsequent event-related potential (ERP) research was conducted; the results revealed that the neural response to audiovisual stimuli in older adults was stronger than that in younger adults (Diaconescu et al., 2013). The stronger neural response in older adults was confirmed in the medial prefrontal and inferior parietal regions 100 ms after stimulus onset (Diaconescu et al., 2013). These studies supported the idea that the enhanced audiovisual integration in older adults related to the activation of specific brain regions may compensate for deficiencies in unimodal sensory processing (Laurienti et al., 2006; Davis et al., 2008; Mozolic et al., 2012; Freiherr et al., 2013). Notably, the above studies on audiovisual integration in older adults mostly presented a simple combination of auditory and visual stimuli and were based on non-speech paradigms. However, findings regarding improved audiovisual integration in older adults were equivocal when focusing on speech perception, which is more complex and requires greater cognitive resources (Freiherr et al., 2013; Stevenson and Wallace, 2013). Sommers et al. (2005) examined the age-related difference in audiovisual integration with speech stimuli by testing the contribution of visual and auditory processing to audiovisual integration. The results showed that the audiovisual gain in older adults less than that in younger adults. The authors further proposed that the encoding of visual information was reduced in older adults, which may diminish their ability to comprehend auditory information (Sommers et al., 2005). In contrast, other researchers reported that audiovisual speech processing did not deteriorate in older adults. That is, older adults exhibited equivalent audiovisual gain with younger adults, with a larger reduction in the amplitude of the N1 component in the audiovisual condition relative to the summed amplitude in the auditory and visual conditions (Winneke and Phillips, 2011). This difference in neural alteration of older adults in audiovisual speech processing might be due to accumulated experience, suggesting that older adults would be better at extracting useful information from visual stimuli (lip movements) to predict the upcoming auditory stimuli (spoken utterance) (Winneke and Phillips, 2011). These results suggested that the discrepancy in audiovisual integration between older and younger adults during audiovisual speech perception remains controversial. It remains to be determined whether, in older adults, sensory processing mainly affects audiovisual integration leading to reduced audiovisual integration compared to younger adults or whether prior experience influences audiovisual integration leading to similar or enhanced audiovisual integration compared to younger adults. According to the multistage audiovisual integration theory, there is a distinct early stage of integration where separate visual information influences the auditory processing at sensory level and a later stage of integration where top-down process engages. The early stage of integration was governed by sensory processing efficiency and later stage of integration was influenced by top-down factors (i.e., prior experience or top-down attention). The relation between increasing age and the functioning of sensory processing and top-down processing turns to be different trend. Despite older adults undergoes age-related decline in sensory processing efficiency, there is a dissociation in top-down processing. That is older adults have top-down deficits but have more knowledgeable experience. Given the above reasons, we hypothesized that the audiovisual integration at early and later stage between older adults and younger adults were different. Therefore, we examined different stages of audiovisual integration with ERPs and compared the neural activity during the processing of audiovisual speech stimuli between age groups.

Additionally, previous researchers have found that stimulus intensity impacts audiovisual integration (Senkowski et al., 2011; Mozolic et al., 2012). Many behavioral studies have shown that the magnitude of audiovisual integration is inversely associated with stimulus intensity. That is, audiovisual integration is greater with low-intensity stimuli than high-intensity stimuli (Corneil et al., 2002). Subsequent research using ERPs demonstrated a robust audiovisual integration effect in the right anterior and left posterior regions, particularly under low-intensity conditions compared to middle-intensity and high-intensity conditions (Senkowski et al., 2011). This phenomenon was considered as inverse effectiveness (IE) (Stein and Stanford, 2008). Numerous aging researches indicated that inverse effectiveness differs according to speech stimuli. Several behavioral studies have shown that audiovisual integration in older adults at the word and sentence levels is not enhanced in low-intensity conditions compared to high-intensity conditions, suggesting that IE in older adults is not found when processing complex speech stimuli (Gordon and Allen, 2009; Tye-Murray et al., 2010; Stevenson et al., 2015; Jansen et al., 2018). However, conflicting results were reported with relatively simple speech stimuli, at the phoneme level. Using phonemes as stimuli, Tye-Murray et al. (2011) reported that older adults only showed audiovisual enhancement when the stimuli intensity was relatively high. When the signal contrast was reduced (low-intensity stimuli), such enhancement was not found (Tye-Murray et al., 2011). This finding suggests that older adults are less able to combine information from visual and auditory modalities when stimulus intensity is lower. However, some researchers have suggested that audiovisual processing is preserved at more elementary levels of speech perception (Stevenson et al., 2015). That is, audiovisual integration in older adults increases as stimulus intensity decreases at the phoneme level (Stevenson et al., 2015). These conflicting findings indicate instability in IE during audiovisual integration at the phoneme level in older adults. In addition, a speech perception task administered during functional magnetic resonance imaging (fMRI) suggested that the superior temporal sulcus (STS) is strongly associated with IE, given the larger BOLD activations in the STS due to audiovisual speech stimuli compared to unisensory speech stimuli in the low-intensity conditions (Stevenson and James, 2009). Due to the unstable IE and the deterioration of the STS in older adults (de Dieuleveult et al., 2017), explorations of IE related to audiovisual integration at the phoneme level is necessary in older adults. Therefore, we compared the magnitude of audiovisual integration between high-intensity conditions and low-intensity conditions in older and younger adults during a speech discrimination task based on phoneme-level stimuli. Evidence showed that there is a direct link between sensory processing and higher-order cognitive processing, reflected by the factor that they may share same resources (Schneider and Pichorafuller, 2000). Speech perception at low-intensity condition is more effortful for older adults. We hypothesized that if sensory processing is effortful, more processing resources have to be devoted to sensory processing, which may lead to enhanced audiovisual integration. In return, this leads to fewer resources available for high-order cognitive processing, which may result in reduced audiovisual integration at this stage.

## Materials and methods

### Participants

Twenty younger adults (13 females, mean age = 21.3 years, SD = 1.9) and 20 older adults (13 females, mean age = 63.5 years, SD = 3.1) were recruited for the present study. All participants were right-handed and reported having normal or corrected-to-normal vision and hearing capabilities. In addition, the Mini-Mental State Examination (MMSE) was used to screen for cognitive impairments in the older participants. This study was approved by the Ethics Committee of Hubei University, and informed consent was acquired from each participant. All participants were given a reward after the procedures were completed.

### Stimuli and procedure

Audiovisual stimuli were created from audio-video recordings of a Chinese woman uttering “ga” and “ba” using Huawei Honour View 10. Unimodal stimuli were created by separating the video clips and audio tracks from the audiovisual recording using Premiere Pro Cc 2018. Under the unimodal visual condition, the video clips were presented in silence, whereas under the unimodal auditory condition, the audio clips were presented without visual information. The stimuli were presented with real audiovisual onset delay. Specifically, the auditory speech started 700 ms (“ba”) with a duration of 300 ms, 680 ms (“ga”) with a duration of 320 ms relative to the beginning of the videos. The lip movement (“ga”) started 370 ms relative to the beginning of the video and the lip movement (“ba”) started 350 ms relative to the beginning of the video. The duration of the lip movement (“ga”) was 630 ms while that of lip movement (“ba”) was 650 ms.

**FIGURE 1:**
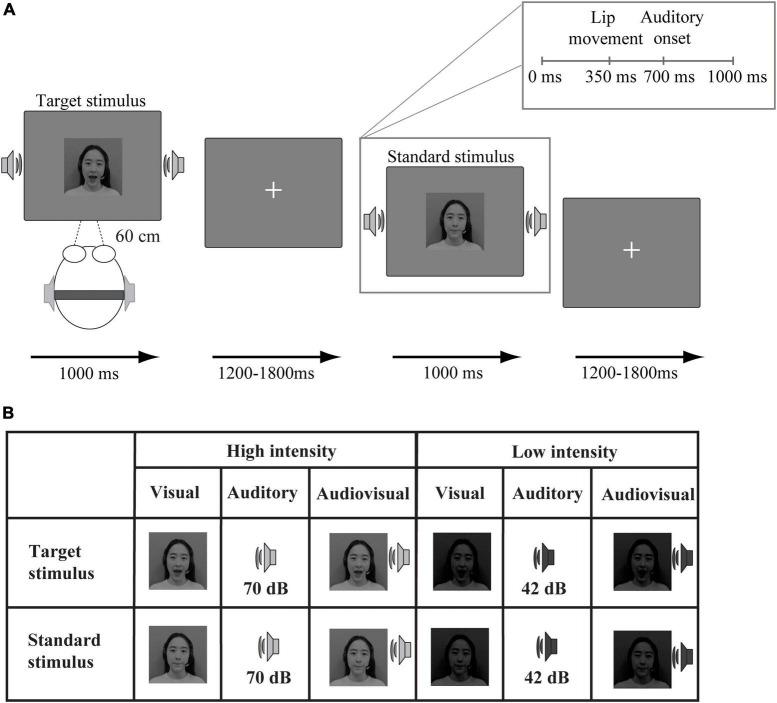
Schematic illustration of the experimental procedure. **(A)** An example of an audiovisual target stimulus and an audiovisual standard stimulus. **(B)** Twelve stimulus types were presented: target (“ga”) and standard (“ba”) stimuli presented in visual, auditory, and audiovisual modalities under high-intensity and low-intensity conditions.

In addition, the visual stimuli were obtained at a resolution of 996 × 554 pixels and later converted to 720 × 480 pixels. The auditory stimuli were 32-bit and had a sampling rate of 44.1 kHz. Michelson contrast, (max − min)/(max + min), was adopted to manipulate the intensity of visual stimuli. The high- and low-intensity stimuli had 90 and 10% contrast, respectively. The intensity of auditory stimuli was manipulated by the sound pressure level. Specifically, we conducted a pre-experiment (stimuli detecting task) prior to all experiments to determine the auditory stimuli intensity. The auditory stimulus at 70 dB was considered as high-intensity because the accuracy is 90% when detecting it. The auditory stimulus was decreased to 42 dB (70% accuracy), which was considered as low-intensity, see Figure 1B.

In the formal experiment, the stimuli were divided into two types (target and standard). The visual target stimulus was the lip movements associated with “ga,” and the standard stimulus was the lip movements associated with “ba.” The auditory target stimulus was the sound “ga,” and the standard stimulus was the sound “ba.” Additionally, the audiovisual target stimuli were the combination of the visual and auditory target stimuli. The audiovisual standard stimuli were the combination of the visual and auditory standard stimuli.

The experiment included 3 high-intensity blocks and 3 low-intensity blocks. Each block consisted of 15 target stimuli (5 auditory, 5 visual, and 5 audiovisual) and 90 standard stimuli (30 auditory, 30 visual, and 30 audiovisual). The experiment was presented via E-prime 2.0. As shown in Figure 1, visual stimuli were displayed on a 19-inch Dell screen at a distance of 60 cm from the participant, and auditory stimuli were presented via earphones. The target or standard stimuli from three modalities (visual, auditory, and audiovisual) were randomly presented for 1,000 ms, see Figure 1A. The interstimulus interval ranged between 1,200 and 1,800 ms. The experiment was conducted in a sound-attenuated room. Participants were required to fix their eyes on the central of the monitor in which the fixation presented during presentation. The task was to press the left mouse button as quickly and accurately as possible to indicate the target stimulus “ga” (unimodal or bimodal) but not to press the button in response to the standard stimulus “ba” (unimodal or bimodal). After completing each block, participants were allowed to take a 1-min break.

### Data analysis

#### Behavioral data

Before analyzing the behavioral data, reactions were excluded if they were three standard deviations from the mean. The mean number of excluded trials in older adults was 0.65 (high-intensity: visual = 1, auditory = 0.56, audiovisual = 0.22; Low-intensity: visual = 0.73, auditory = 1.12, audiovisual = 0.22). The mean number of remaining trials in younger adults was 0.18 (high-intensity: visual = 0.06, auditory = 0.22, audiovisual = 0.17; low-intensity: visual = 0.22, auditory = 0.17, audiovisual = 0.22). One younger participant and two older participants were excluded because of their poor performance detecting visual stimuli (hit rates lower than 50%). The data from 19 younger adults and 18 older adults were used for further analysis. The hit rates and mean response times to the targets were computed for both younger adults and older adults under low-intensity and high-intensity conditions. We utilized a three-factor analysis of variance (ANOVA) with age as a between-subjects factor and modality and intensity as within-subject factors (if Mauchly’s sphericity was violated, Greenhouse–Geisser corrections were applied to correct the degrees of freedom). The *post-hoc* analysis used pairwise comparison with Bonferroni correction.

#### Event-related potential data

Electroencephalogram (EEG) signals were recorded by an EEG system (BrainAmp MR plus, Gilching, Germany) through 32 electrodes located according to the international 10–20 system using an electrode cap (Easy Cap, Herrsching Breitbrunn, Germany). All signals were referenced to the FCz electrode. Electrooculogram (EOG) data were recorded by two additional electrodes. Horizontal eye movements were recorded from an electrode placed at the outer canthus of the left eye, and vertical eye movements were recorded from an electrode placed approximately 1 cm below the right eye. The impedances of all electrodes were maintained below 5 kΩ.

Electroencephalogram data was stored digitally for offline analysis and was conducted by using Brain Vision Analyser software, including rereferencing to the average of TP9 and TP10. The EEG signals were bandpass filtered from 0.01 to 60 Hz at a sampling rate of 250 Hz. The ERPs elicited by standard stimuli were analyzed as in previous studies (Yang et al., 2021). The ERP epochs in three conditions (visual, auditory, and audiovisual) were locked to auditory onset. Then, the data were epoched from 100 ms before to 600 ms after the onset of the stimuli, and baseline corrections were made from −100 to 0 ms relative to stimulus onset. In addition to the fact that EEG epochs containing eye blinks and small artifacts were removed based on a trail-by-trail inspection of the data, epochs contaminated with large artifacts were rejected from the subsequent averaging if the voltage exceeded ±100 μV at any electrode site relative to baseline. Then, all the remained data without artifacts were averaged for each participant, each stimulus type (auditory, visual, and audiovisual), each stimulus intensity, following digital filtering with a bandpass filter of 0.01–30 Hz. The grand-averaged data were obtained across all the conditions and groups. The mean number of rejected epochs in older adults was 14.30 (high-intensity: visual = 13.56, auditory = 14.28, audiovisual = 13.39; Low-intensity: visual = 14.89, auditory = 14.61, audiovisual = 15.06). The mean number of rejected epochs in younger adults was 13.89 (high-intensity: visual = 16.37, auditory = 13.53, audiovisual = 13.95; Low-intensity: visual = 11.90, auditory = 12.74, audiovisual = 14.84). For remained epochs, the mean number in older adults was 75.70 (high-intensity: visual = 76.44, auditory = 75.72, audiovisual = 76.61; Low-intensity: visual = 75.11, auditory = 75.39, audiovisual = 74.94). The mean number in younger adults was 76.11 (high-intensity: visual = 73.63, auditory = 76.47, audiovisual = 76.05; Low-intensity: visual = 78.10, auditory = 77.26, audiovisual = 75.16). Two older adults and one younger adult were excluded from further analysis due to the loss of more than 70% of the epochs in at least one stimulus type (the excluded participants were identical to those in behavioral data).

To assess the audiovisual integration in ERPs, we calculated the result of ERP(AV) − [ERP(A) + ERP(V)] by subtracting the summed ERPs on the unimodal visual and auditory trials from the ERPs on the audiovisual trials at each time bin for each electrode (Giard and Peronnet, 1999). Then, one-sample *t*-tests were conducted to compare the amplitude of AV − (A + V) with 0 in each time bin ranging from 0 to 600 ms. If more than 7 consecutive data points were significant (α < 0.05), the participant was considered to display audiovisual integration (Senkowski et al., 2011). In the present data, audiovisual integration was defined as at least 7 or more consecutive data points that met an alpha criterion of 0.05, which suited a Bonferroni correction for multiple comparisons. This criterion meets the strict criteria for assessing reliable results when a large number of *t*-tests are calculated (Guthrie and Buchwald, 1991). Based on the results of the *t*-tests, three-time intervals of integration (200–230 ms, 320–360 ms, and 460–500 ms) and five regions of interest (frontal: Fz, F3, F4, F7, F8; fronto-central: FC1, FC2, FC5, FC6; central: C3, C4, Cz; centro-parietal: CP1, CP2, CP5, CP6; occipital: O1, O2, Oz) were chosen for further analysis. In addition, ERPs were averaged across electrodes within each region of interest. For each of these time intervals (200–230 ms, 320–360 ms, and 460–500 ms), we conducted a repeated-measures ANOVA with the mean amplitude of ERP(AV) − [ERP(A) + ERP(V)] as the dependent variable. The ANOVA included the between-subject factor of age and the within-subject factors of stimuli intensity and region of interest (ROI). Greenhouse–Geisser corrections were applied to correct the degrees of freedom if the sphericity assumption was violated. The *post-hoc* analysis used pairwise comparison with Bonferroni correction. All statistical analyses were conducted by SPSS version of 26.0 software package.

## Results

### Behavioral results

#### Hit rates

The results showed a main effect of modality [*F*_(2_, _70)_ = 4.36, *p* = 0.02, η_*p*_^2^ = 0.11]; specifically, audiovisual targets (*M* = 98%, SE = 0.3%) detected correctly at a significantly higher rate than either unimodal visual targets (*M* = 94%, SE = 0.8%) or auditory targets (*M* = 95%, SE = 1.3%). There was also a significant main effect of age [*F*_(1_, _35)_ = 6.25, *p* = 0.02, η_*p*_^2^ = 0.15], as younger adults (*M* = 98%, SE = 0.8%) exhibited a significantly higher hit rate than older adults (*M* = 94%, SE = 1%). Additionally, we found a significant effect of intensity [*F*_(1_, _35)_ = 4.58, *p* = 0.04, η_*p*_^2^ = 0.15], suggesting that high-intensity targets (*M* = 97%, SE = 0.5%) were detected correctly at a significantly higher rate than low-intensity targets (*M* = 95%, SE = 1%). However, the interaction of these three factors was not significant [*F*_(2_, _70)_ = 0.44, *p* > 0.05, η_*p*_^2^ = 0.01]. Additionally, the intensity × age, modality × age, and intensity × modality interactions were not significant [*F*_(1_, _35)_ = 0.25, *p* > 0.05, η_*p*_^2^ = 0.01], [*F*_(2_, _70)_ = 0.34, *p* > 0.05, η_*p*_^2^ = 0.01], and [*F*_(2_, _70)_ = 0.86, *p* > 0.05, η_*p*_^2^ = 0.02], respectively. The detailed hit rates were demonstrated in Table 1.

**TABLE 1:** Mean response times and hit rates for younger and older adults under high-intensity and low-intensity conditions.

	Low intensity			High intensity		
	Visual	Auditory	Audiovisual	Visual	Auditory	Audiovisual
**Older adults**						
HT (%)	92 (11)	92 (15)	96 (3)	94 (6)	93 (8)	98 (3)
RT (ms)	778 (205)	1,021 (175)	712 (158)	804 (208)	1004 (161)	724 (180)
**Younger adults**						
HT (%)	94 (9)	96 (5)	99 (2)	97 (4)	98 (2)	99 (1)
RT (ms)	481 (150)	821 (126)	468 (158)	409 (145)	780 (104)	412 (137)

Standard deviations are presented in parentheses.

#### Response times

The results revealed that the main effect of modality was significant [*F*_(2_, _70)_ = 212.51, *p* < 0.001, η_*p*_^2^ = 0.86]; specifically, the response time to audiovisual stimuli (*M* = 579 ms, SE = 25 ms) was significantly shorter than that to visual (*M* = 618 ms, SE = 28 ms) and auditory stimuli (*M* = 907 ms, SE = 23 ms) alone. The main effect of age was also significant [*F*_(1_, _35)_ = 36.17, *p* < 0.001, η_*p*_^2^ = 0.51], indicating that the response time of the older adults (*M* = 840 ms, SE = 33 ms) was longer than that of the younger adults (*M* = 562 ms, SE = 32 ms). However, there was no significant main effect of intensity [*F*_(1_, _35)_ = 3.54, *p* > 0.05, η_*p*_^2^ = 0.09].

In addition, we observed a two-way interaction between age and stimulus intensity [*F*_(1_, _35)_ = 5.98, *p* = 0.02, η_*p*_^2^ = 0.15]. Therefore, further *post-hoc* analyses were conducted. Specifically, regarding age, we found that older adults (*M* = 844 ms, SE = 33 ms) responded significantly slower than younger adults (*M* = 534 ms, SE = 32 ms) under the high-intensity condition [*t*(35) = 6.67, *p* < 0.001]. Similar results were found under the low-intensity condition, with the reaction time for older adults (*M* = 837 ms, SE = 35 ms) being significantly longer than that for younger adults (*M* = 590 ms, SE = 34 ms) [*t*(35) = 4.97, *p* < 0.001]. Regarding stimulus intensity factor, there was no significant difference in response times between the high-intensity (*M* = 844 ms, SE = 33 ms) and low-intensity conditions (*M* = 837 ms, SE = 35 ms) in older adults [*t*(17) = 0.41, *p* > 0.05]. However, younger adults responded to high-intensity stimuli (*M* = 534 ms, SE = 32 ms) significantly faster than to low-intensity stimuli (*M* = 590 ms, SE = 34 ms) [*t*(18) = −3.02, *p* < 0.01]. There was no three-way interaction [*F*_(2_, _70)_ = 2.62, *p* = 0.09, η_*p*_^2^ = 0.07]. The detailed response times were shown in Table 1.

### Event-related potential results

#### Audiovisual integration at 200–230 ms

The ANOVA results showed no main effects of intensity, ROI or age [*F*_(1_, _35)_ = 3.82, *p* > 0.05, η_*p*_^2^ = 0.10], [*F*_(4_, _140)_ = 1.94, *p* > 0.05, η_*p*_^2^ = 0.06], and [*F*_(1_, _35)_ = 1.29, *p* > 0.05, η_*p*_^2^ = 0.04], respectively.

**FIGURE 2:**
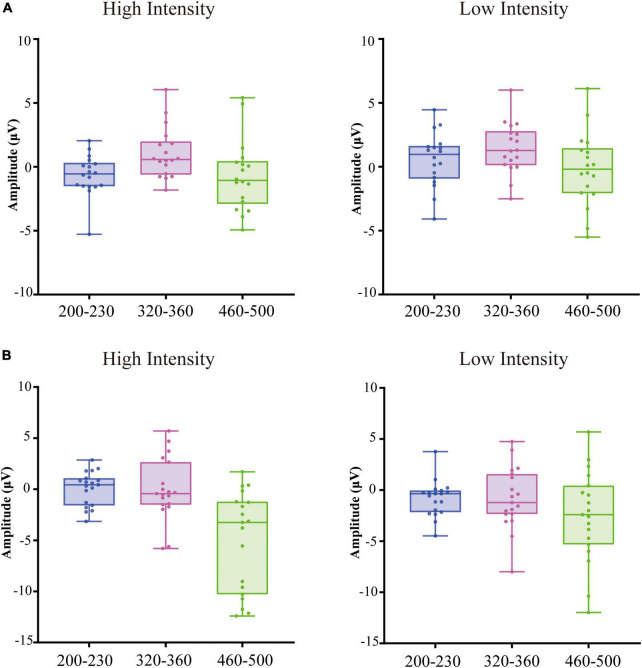
Box plots of the amplitude averaged over frontal, fronto-central, central, centro-parietal, Occipital regions at 200–230 ms relative to auditory onset, 320–360 ms relative to auditory onset, 460–500 ms relative to auditory onset in older adults **(A)** and younger adults **(B)**.

**FIGURE 3:**
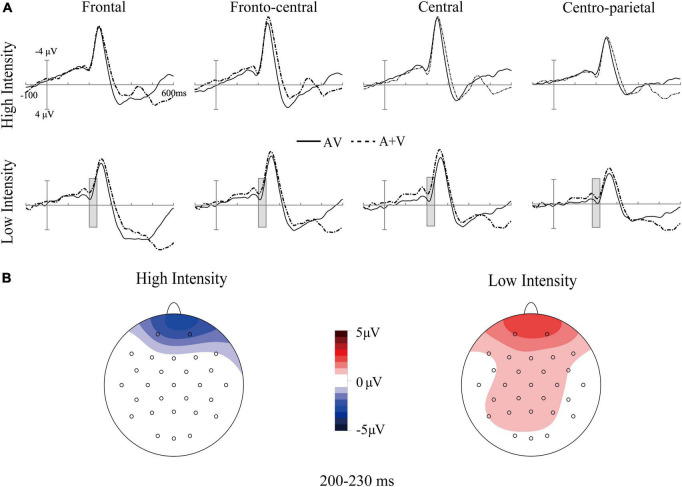
Grand-average event-related potentials of audiovisual stimuli (AV) and the sum of visual and auditory stimuli (A + V) from 100 ms before the stimuli to 600 ms after stimulus onset **(A)**, as well as the topographic map of audiovisual integration [AV–(A + V)] **(B)** in older adults under high- and low-intensity conditions. The gray cubes represent the integration time window at 200–230 ms relative to auditory onset, mainly illustrating inverse effectiveness in older adults.

**FIGURE 4:**
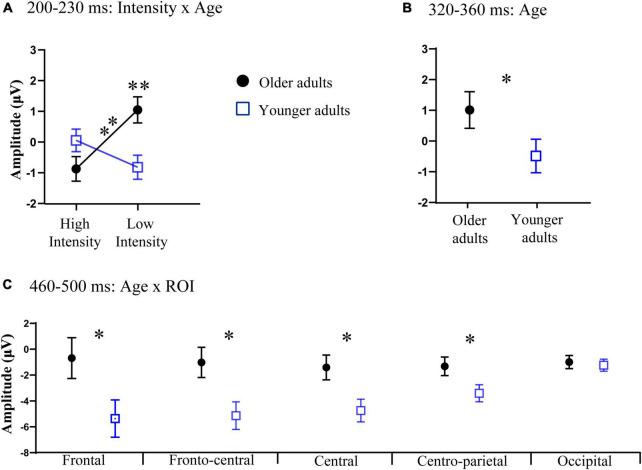
**(A)** Significant interaction between intensity and age at 200–230 ms relative to auditory onset. **(B)** Significant main effect of age at 320–360 ms relative to auditory onset. **(C)** Significant interaction between age and the region of interest (ROI) at 460–500 ms relative to auditory onset. Error bars are the standard error of the mean (SEM); *p < 0.05, **p < 0.01.

Additionally, the three-way interaction was also not significant [*F*_(4_, _140)_ = 1.11, *p* > 0.05, η_*p*_^2^ = 0.03]. However, the analysis showed a significant interaction between ROI and age [*F*_(4_, _140)_ = 3.31, *p* = 0.05, η_*p*_^2^ = 0.07]. *Post-hoc* analysis indicated that the amplitude in the frontal region of older adults (*M* = 0.68 μV, SE = 0.44 μV) was significantly greater than that of younger adults (*M* = −0.52 μV, SE = 0.41 μV) [*t*(35) = 2.01, *p* = 0.05]. The interaction between intensity and age was also significant [*F*_(1_, _35)_ = 13.64, *p* < 0.001, η_*p*_^2^ = 0.29]. *Post-hoc* analysis indicated that the amplitude under the low-intensity condition (*M* = 1.05 μV, SE = 0.43 μV) was more positive than that under the high-intensity condition (*M* = −0.87 μV, SE = 0.40 μV) in older adults [*t*(17) = −4.61, *p* < 0.001], see Figures 2, 3. However, such an effect was not found in younger adults (high-intensity: *M* = 0.10 μV, SE = 0.37 μV, low-intensity: *M* = −0.82 μV, SE = 0.39 μV) [*t*(18) = 1.46, *p* > 0.05]. Moreover, in terms of age factor, greater amplitude was also found in older adults (*M* = 1.05 μV, SE = 0.43 μV) under the low-intensity condition comparing to younger adults (*M* = −0.82 μV, SE = 0.39 μV) [*t*(35) = 3.23, *p* < 0.01]. There was no significant difference between younger (*M* = 0.10 μV, SE = 0.37 μV) and older adults (*M* = −0.87 μV, SE = 0.40 μV) under the high-intensity condition [*t*(35) = −1.72, *p* > 0.05], see Figure 4A.

#### Audiovisual integration at 320–360 ms

**FIGURE 5:**
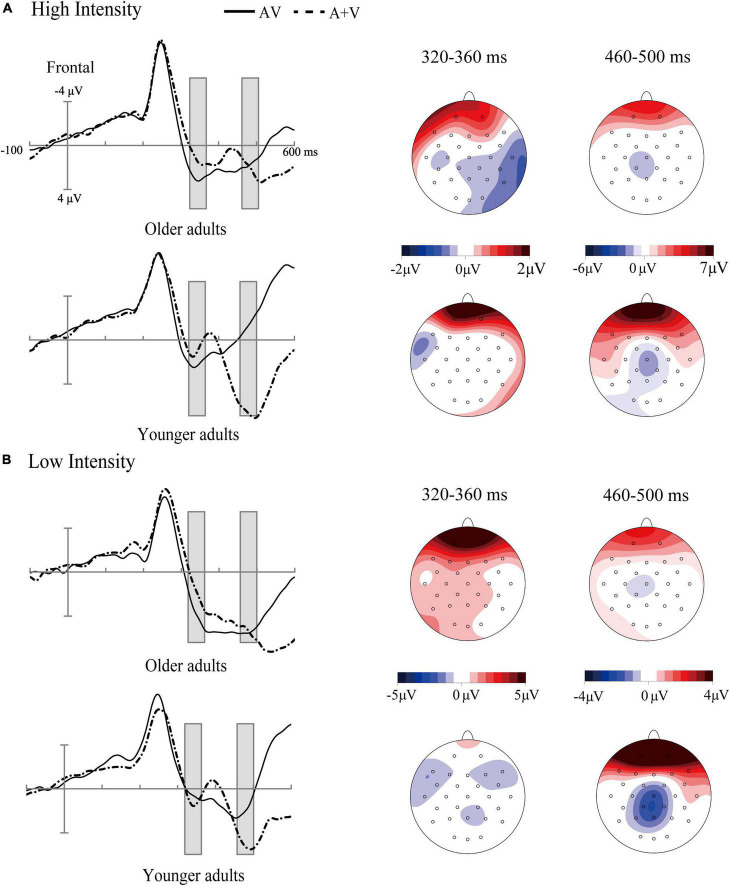
Grand-average event-related potentials of audiovisual stimuli (AV) and the sum of visual and auditory stimuli (A + V) from 100 ms before the stimuli to 600 ms after stimulus onset as well as the topographic map of audiovisual integration [AV–(A + V)] in different age groups under high-intensity **(A)** and low-intensity conditions **(B)**. The gray cubes represent the integration time windows at 320–360 ms relative to auditory onset and 460–500 ms relative to auditory onset, illustrating the difference in audiovisual integration between older and younger adults.

In this time window, we found that there was a significant main effect of age [*F*_(1, 35)_ = 5.59, *p* = 0.02, η_*p*_^2^ = 0.15], indicating that the amplitude in older adults (*M* = 1.00 μV, SE = 0.48 μV) was more positive than that of younger adults (*M* = −0.54 μV, SE = 0.44 μV), see Figures 4B, 5. The main effect of ROI was also significant [*F*_(4, 140)_ = 9.04, *p* < 0.001, η_*p*_^2^ = 0.22], with the amplitude of the frontal region (*M* = 1.37 μV, SE = 0.56 μV) being significantly more positive than that in the central (*M* = −0.42 μV, SE = 0.38 μV) and centro-parietal (*M* = −0.37 μV, SE = 0.29 μV) and fronto-central regions (*M* = 0.44 μV, SE = 0.42 μV). The amplitude of the fronto-central region (*M* = 0.44 μV, SE = 0.42 μV) was greater than that in the central (*M* = −0.42 μV, SE = 0.38 μV) and centro-parietal regions (*M* = −0.37 μV, SE = 0.29 μV). The main effect of intensity was not significant [*F*_(1, 35)_ = 0.18, *p* > 0.05, η_*p*_^2^ = 0.01].

The three-way interaction was not significant [*F*_(4, 140)_ = 1.27, *p* > 0.05, η_*p*_^2^ = 0.04]. The interaction between intensity and age was also not found [*F*_(1, 35)_ = 3.89, *p* = 0.057, η_*p*_^2^ = 0.11].

#### Audiovisual integration at 460–500 ms

The ANOVA results showed that the main effect of intensity was not significant [*F*_(1, 35)_ = 2.91, *p* > 0.05, η_*p*_^2^ = 0.08]. There was a significant main effect of ROI [*F*_(4, 140)_ = 3.37, *p* = 0.01, η_*p*_^2^ = 0.10], with the amplitude of central region (*M* = −3.16 μV, SE = 0.65 μV) being more negative than that in the centro-parietal (*M* = −2.56 μV, SE = 0.49 μV) and occipital regions (*M* = −1.37 μV, SE = 0.35 μV); additionally, the amplitude of fronto-central (*M* = −3.14 μV, SE = 0.79 μV) and centro-parietal (*M* = −2.56 μV, SE = 0.49 μV) regions was more negative than that in the occipital region (*M* = −1.37 μV, SE = 0.35 μV). A significant main effect of age was found [*F*_(1, 35)_ = 6.98, *p* = 0.01, η_*p*_^2^ = 0.17], with a more negative amplitude in younger adults (*M* = −3.56 μV, SE = 0.89 μV) than in older adults (*M* = −1.71 μV, SE = 0.78 μV), see Figure 5.

The three-way interaction was not significant [*F*_(4, 140)_ = 2.15, *p* > 0.05, η_*p*_^2^ = 0.06]. However, we found a significant interaction between ROI and age [*F*_(4, 140)_ = 9.06, *p* < 0.001, η_*p*_^2^ = 0.21]. *Post-hoc* analysis revealed that the amplitudes were more negative in the frontal (*M* = −5.37 μV, SE = 1.44 μV), fronto-central (*M* = −5.19 μV, SE = 1.07 μV), central (*M* = −4.83 μV, SE = 0.88 μV), centro-parietal (*M* = −3.6 μV, SE = 0.66 μV) regions of younger adults than those in older adults (frontal: *M* = −0.49 μV, SE = 1.58 μV, fronto-central: *M* = −1.08 μV, SE = 1.16 μV, central: *M* = −1.49 μV, SE = 0.96 μV, centro-parietal: *M* = −1.25 μV, SE = 0.51 μV) [*t*(35) = 2.28, *p* < 0.05; *t*(35) = 2.60, *p* < 0.05; *t*(35) = 2.57, *p* < 0.05; *t*(35) = 2.13, *p* < 0.05;], see Figure 4C.

## Discussion

The current study compared audiovisual integration in older and younger adults under high- and low-intensity conditions. The results showed enhanced audiovisual integration at 320–360 ms in older adults compared to younger adults, regardless of stimulus intensity and ROI. Conversely, at a relatively later stage (460–500 ms), attenuated audiovisual integration in the frontal, fronto-central, central, and centro-parietal regions were found in the older adults compared to younger adults. In addition, in older adults, we found greater audiovisual integration at 230–300 ms under the low-intensity condition than the high-intensity condition, suggesting inverse effectiveness occurred. However, inverse effectiveness was not observed in younger adults.

### Effect of aging on audiovisual integration

At 320–360 ms, older adults exhibited greater audiovisual integration than younger adults. The weaker ability of suppressing crossmodal information in older adults may render a plausible explanation for the results (de Dieuleveult et al., 2017). Previous study utilized a cued audiovisual discrimination task to explore changes in audiovisual integration under selective and divided conditions between age groups (Hugenschmidt et al., 2009a). The results not only showed that older adults had greater audiovisual integration under selective conditions but also that older adults exhibited greater audiovisual integration under divided conditions than younger adults (Hugenschmidt et al., 2009a). This finding might be because younger adults could ignore modality-specific irrelevant information, which suppressed audiovisual integration. In contrast, older adults may have been unable to judge whether the information was irrelevant effectively, leading to enhanced audiovisual integration. Moreover, their subsequent study found neural evidence that older adults showed greater brain activity than younger adults when they were involved in irrelevant information (Hugenschmidt et al., 2009b). From this neural evidence along with our own behavioral data indicating that older adults achieved lower accuracy (Table 1), we infer that the greater audiovisual integration of older adults reflected greater distraction from irrelevant information. This phenomenon was also found in other audiovisual perception tasks, such as the McGurk task or the sound-induced illusion task, in which the older adults had a higher possibility of being influenced by the interaction between visual modality and auditory modality and was more vulnerable to yield the audiovisual effect (DeLoss et al., 2013; Sekiyama et al., 2014).

Because overall good performance was found in both groups, indicating that older adults can achieve a level of performance comparable to that of younger adults. This could be explained by compensatory mechanisms, which help maintain good performance in older adults (Cabeza et al., 2002; Diaconescu et al., 2013; Freiherr et al., 2013). In other words, increased brain activity in some regions may compensate the older adults cognitive decline to improve their performance. Moreover, Enhanced activation in older adults was relative to younger adults. The compensatory interpretation is often invoked when older adults show more activity in a brain region than younger adults when they perform the same task (Cabeza et al., 2002). If we find enhanced activation in older adults comparing to younger adults and their behavioral performance was also preserved comparing to younger adults, the compensatory mechanisms may work (Davis et al., 2012). Some studies have further confirmed that enhanced activation in frontal and posterior parietal areas is positively associated with the performance of speech perception in older adults, suggesting a compensatory role in aiding older adults to achieve better performance (Wong et al., 2009). However, Reuter-Lorenz and Cappell (2008) proposed that the compensatory mechanism is effective at lower levels of task demand. When task-demanding increases at high levels of the task, the cognitive resources may reach limitation, resulting in insufficient processing and poor performance in older adults (Rypma et al., 2007; Reuter-Lorenz and Cappell, 2008). For instance, studies utilizing working memory tasks have shown greater activation in the prefrontal and parietal cortices in older adults than younger adults under relatively low working memory loads; conversely, less activity in these areas in older adults was observed under high working memory loads (Schneider and Pichorafuller, 2000; Mattay et al., 2006). In terms of speech stimuli, the findings of the current study, which used relatively elementary speech stimuli (i.e., phonemes), were consistent with those of a previous study, which reported that audiovisual processing was intact at low levels of speech perception in older adults (Stevenson et al., 2015). In other words, the compensatory mechanism might have worked in older adults due to the elementary speech stimuli we used.

Despite well-documented increases in audiovisual integration related to speech stimuli in older adults (Cienkowski and Carney, 2002), we found attenuated audiovisual integration in the frontal, fronto-central, central, and parietal regions in older adults compared to younger adults at 460–500 ms. Xi et al. (2020) suggested that different integration time windows may reflect different stages of processing during audiovisual integration (Xi et al., 2020). These authors investigated the neural activity associated with audiovisual integration in different attention conditions. During the experiment in their studies, participants were required to focus on the stimuli on one side while ignoring the stimuli on the opposite side, which produced two types of attention conditions. Under attended condition, when the participants were asked to respond to stimuli on one side (e.g., left session), the stimuli were presented on the same side (e.g., left side). Under unattended condition, for instance, the participants were asked to respond to stimuli on right side (right session), the stimuli were presented on left side. Specifically, in the attended condition, audiovisual integration was observed in the frontal regions at 560–600 ms. In the unattended condition, audiovisual integration was observed in the centro-frontal regions at 340–360 ms. The authors suggested that audiovisual integration (340–360 ms) in the unattended condition was related to automatic processes (Xi et al., 2020). More importantly, a goal-driven process influenced by top-down attention was located at a later audiovisual integration time window (560–600 ms) in the attended condition (Xi et al., 2020). In the present study, participants were asked to attend to the speech stimuli and to carefully focus on the speaker’s lip movements and utterances to discriminate which stimuli to respond to, suggesting that the task was under attended condition. Therefore, we infer that later audiovisual integration at 460–500 ms in our research may reflect the influence of top-down attention processing. Additionally, previous studies have shown that the frontal and fronto-central regions are highly associated with cognitive function, such as top-down attention (de Dieuleveult et al., 2017). We infer that deficit in top-down attention may offer an interpretation for older adults having attenuated audiovisual integration in these regions compared to younger adults.

### Inverse effectiveness in audiovisual integration

Interestingly, N200 component could be observed at 200–230 ms (see Figure 3), which may reflect the auditory attentional processes during audiovisual speech processing (Tremblay et al., 2021). However, our main concern is the time window of audiovisual integration similar to previous study (Ren et al., 2018). At 200–230 ms, enhanced audiovisual integration in older adults was observed when the presented stimuli were in the low-intensity condition, which reflects the involvement of higher processing resources. The theory of cognitive resource limitations describes the mutual constraint relationship between sensory and cognitive processing (Schneider and Pichorafuller, 2000). In particular, sensory and cognitive processing share common resources from unified information-processing systems, indicating that alterations in either processing would influence the other (Schneider and Pichorafuller, 2000). When stimuli intensity decreases, older adults may not effectively perceive the degraded information delivered by the stimuli. More processing resources may have been recruited to engage in this stage at the cost of the processing resources allocated to cognitive processing, resulting in greater audiovisual integration in older adults under low-intensity conditions.

Additionally, according to predictive coding theory, our perception is determined by a trade-off between prediction based on prior experience accumulated over our life span and efficiency of sensory processing (Sohoglu et al., 2012; Alm and Behne, 2013; Chan et al., 2017). However, a Bayesian model suggests that the weight between predictions and efficiency of sensory processing changes with age (Chan et al., 2017). Some investigations using sound-induced flash tasks have attempted to explore differences in the weighting of predictions and sensory efficiency in older and younger adults. Their results showed that older adults had increased beta-band activity before processing the stimulus, indicating that older adults may rely more heavily on predictions based on prior experience (Chan et al., 2017). In the current study, visual speech information could play a crucial role in this predictive process. Because in many cases, lip movements are visible which may set up predictions about what is about to be heard. The prediction based on visual information (lip movements) may depend on the prior experience. Studies have shown that audiovisual speech integration is a skill that is obtained by experiencing sound and observing lip movements (Dick et al., 2010). These experiences could contribute to the development of audiovisual speech (Dick et al., 2010). As the amount of information accumulated in our priors increases throughout one’s lifetime, older adults could make use of experience to perceive the low stimuli that delivered insufficient information.

Interestingly, some studies have reported that IE in audiovisual integration occurs at an early stage, indicating that early sensory processing may be associated with IE (Giard and Peronnet, 1999; Senkowski et al., 2011). However, our results showed delayed IE compared with these studies. This dissociation could be explained by additional cognitive processing engaged in audiovisual speech. Previous studies have investigated audiovisual integration at different levels of complexity. At lower levels, researchers have adopted simple stimuli, such as white noise or flashing lights, to elicit audiovisual integration. These stimuli can be integrated due to the factor that they coincide with regard to time, suggesting that only interactions of sensory processing occurred. At higher levels, such as audiovisual speech, speech stimuli from different modalities may still have a temporal relationship, but may also have a connection beyond the temporal factor (Wyk et al., 2010). That is, the speech sounds and lip movements are not only related in time, but also related by phonological knowledge. The current study showed that delayed IE might reflect phonological knowledge processing rather than sensory processing.

Our discussion was based on the ERP results of audiovisual integration elicited by the approach (ERP(AV) − [ERP(A) + ERP(V)]). The limitations of this approach result in if anticipatory slow are elicited in association with each of the stimulus types (A, V, AV). The potentials of audiovisual integration (ERP(AV) − [ERP(A) + ERP(V)]) is subtracted twice, creating a deflection that might be confounded with true audiovisual integration (Teder-Sälejärvi et al., 2002). Future studies may take this effect into consideration.

## Conclusion

We present results of older and younger adults on an audiovisual discrimination task with varying levels of stimulus intensity. These results confirm differences in the audiovisual integration process between the age groups. Greater audiovisual integration was found in older adults at a relatively earlier stage (320–360 ms). However, due to the deficits of top-down attention in older adults, their audiovisual integration was attenuated at a relatively later stage (460–500 ms). Additionally, the results showed different effects of stimulus intensity in older and younger adults. That is, IE occurred exclusively in older adults. Moreover, our research showed that such an effect was delayed (200–230 ms) compared that found in a previous study regarding IE; this delay might be associated with phonological knowledge processing in audiovisual speech.

## Data availability statement

The raw data supporting the conclusions of this article will be made available by the authors, without undue reservation.

## Ethics statement

The studies involving human participants were reviewed and approved by the Ethics Committee of Hubei University, Wuhan, China. The patients/participants provided their written informed consent to participate in this study.

## Author contributions

WY and ZZ conceived and designed the experiment. XY, ZL, and SL collected the data. AG analyzed the data, wrote the manuscript, and received the comments from YR and JW. HY and JC interpreted the data. All authors contributed to the article and approved the submitted version.
